# Supplementary material for: Credibility assessment of in silico clinical trials for medical devices
Source: PLoS Comput Biol. 2024 Aug 8;20(8):e1012289. doi: 10.1371/journal.pcbi.1012289 (PMC11309390; doi:10.1371/journal.pcbi.1012289)
Supplement: S1 Text — The supplementary material document discusses possible modifications to the example workflow in Section 4 for ISCTs that are different to the case considered in Section 4. (PDF) [file pcbi.1012289.s001.pdf]

Supplementary Material for:
Credibility Assessment of In Silico Clinical Trials for Medical Devices

Pras Pathmanathan, Kenneth Aycock, Andreu Badal, Ramin Bighamian,
Jeff Bodner, Brent A. Craven, Steven Niederer

The example workflow presented in Section 4 was based on the assumptions stated therein on the ISCT being assessed. Suggested modifications to the example workflow for cases different to these assumptions are discussed below.

- Device sub-model:
  - It was assumed that the ISCT uses a physics-based device sub-model. If there is no device sub-model, for example, if a device algorithm is coupled to a patient model, as in Section 2.3.4 and 2.3.7, evidence that the algorithm has been implemented correctly could replace Step 4A, together with evidence to demonstrate it is appropriate to directly couple the algorithm to the patient model.
- Patient and coupled device/patient sub-models:
  - It was assumed the patient model would be validated by creating some subject-specific virtual patients and comparing against data collected from those subjects. However, there are many options for patient model validation as discussed in Section 3.2.1 and Table 2. If an alternative method is used, Steps 4B, 4C, 5B and 5C may need to be adapted.
  - In Step 4B, calculation verification is performed on the uncoupled patient model, and potentially also UQ. These are not included in Step 4C for the coupled device/patient model because the previous results should support the coupled model, except for scenarios where new equations are used to model the coupling (e.g., new interface or boundary conditions such as contact). In such cases, the workflow could be modified to also include calculation verification and/or UQ in Step 4C.
- Other sub-models:
  - It was assumed the clinical outcome model is data-driven and was assessed independently of the other models. However, quantitative information on the clinical outcome model accuracy could be included in the overall Adequacy Assessment in Step 8.
  - It was assumed there is no emulator. If there is an emulator, the emulator error could be assessed and included in the overall Adequacy Assessment in Step 8.
- Virtual cohort:
  - It was assumed the virtual cohort will be composed of synthetic patients created by sampling parameters. This means the only way to validate the full virtual cohort is to perform population-level validation (Section 3.2.3).
    1. If instead the cohort is made up of subject-specific virtual patients, an alternative approach to validating the entire cohort is to repeat Step 4B for every virtual patient (Section 3.2.2, Figure 3a).
    2. For a cohort of synthetic patients, if population-level validation is not feasible, evidence supporting the specific distributions chosen could be generated instead.
