# Supplementary material for: Credibility assessment of in silico clinical trials for medical devices
Source: PLoS Comput Biol. 2024 Aug 8;20(8):e1012289. doi: 10.1371/journal.pcbi.1012289 (PMC11309390; doi:10.1371/journal.pcbi.1012289)
Supplement: Attachment — Submitted filename: response_to_reviewers.pdf [file pcbi.1012289.s002.pdf]

Response to Reviewer Comments for

"Credibility Assessment of In Silico Clinical Trials for Medical Devices"

We would like to thank all three reviewers for their careful readthrough, positive feedback and insightful suggestions for improvements. We believe the revised document is significantly improved. The revised document is attached and highlights new text in red. In response the Reviewer 3's comments, the novel workflow has been moved from the Supplement to the main body. Text that has been moved from supplement is highlighted in blue in the revised paper. Responses to specific concerns are provided below.

Note: one edit was made based on a recent event. The workflow in presented in this paper is a sister workflow to another workflow that we developed for patient-specific models in medical device software, based in our earlier PLOS CompBio paper Galappaththige et al 2022. The sister workflow has just been published available as an FDA Regulatory Science Tool (see here). We reference this sister workflow in two places, lines 727 and 927. In addition, figures were changed to remove icons downloaded from flaticon.com, because the journal informed us their license was incompatible with the journal's. Equivalent open-access icons are now used instead.

Reviewer #1:

This is a very interesting paper with a variety of real-world implications.

Thank you

I think this needs to be clearly stated within the manuscript; there should be a clear statements made about the advantages and disadvantages of this method as well as its application in a real-world setting. What type of medical devices can be used here? there are many different categories and how is this aligned to the current regulatory landscape? simulation methods have been used for a variety of reasons and accepted in some situations. Hence, this should be stipulated within the context of the paper.

Thanks for the suggestion. We have added the following text discussing the advantages and limitations of our method, to the beginning of the Discussion.

- "The workflow we presented offers several advantages over *ad hoc* ISCT evaluation approaches. Firstly, it is systematic, which can reduce planning costs, ensure uniform evaluation of ISCTs, and potentially alleviate stakeholder reliability concerns. Secondly, it is hierarchical, ensuring individual testing of sub-components within the ISCT. This is crucial for ensuring that the overall ISCT model accurately predicts "the right answer for the right reasons" [71]. Thirdly, it is based on the ASME V&V40 [1] approach, making it risk-based. The level of rigor in the credibility activities is selected based on the risk associated with using the model, limiting unnecessary evaluation efforts. Fourthly, it aligns with the framework in the [2], promoting planning and discussion among stakeholders before initiating credibility assessment efforts. This alignment should optimize the process. However, there are some disadvantages to the workflow. It may be

perceived as overly burdensome, and adjustments may be necessary based on the specifics of the ISCT, potentially introducing subjectivity into the process.

Overall, we anticipate that the general approach of the workflow will be applicable to many medical device ISCTs. We believe it should be possible to define a variant of the workflow suitable for each of the example ISCTs reviewed in Section 2.3, except for the Bayesian framework discussed in Section 2.3.6, which differs fundamentally from the others."

What do you mean about acute to long term outcomes? acute and chronic are very specific clinical terms and should not be used in this context as the details written in this section is not really what acute would mean. How would you determine short, medium and long term clinical outcomes? did you try this approach with a specific condition given the complexities linked to communicable and non-communicable diseases.

Thank you. We realized that stating that the clinical outcome model is an 'acute to long-term' model is too restrictive, and, as you state, uses clinical terms too loosely. The clinical outcome model is any model that converts the engineering quantities typically output by the coupled device-patient model (e.g., stresses or strains) into clinical endpoints typically used in a real-world trial. It does not necessarily have map acute parameters to long-term parameters. Therefore, we removed 'acute' and 'long term' from the definition of this model, throughout the paper.

Reviewer #2:

The manuscript provides a compelling exploration of in silico clinical trials (ISCTs) for medical devices, emphasizing in particular the crucial aspect of credibility assessment. This focus is highly relevant in today's context where computational models are increasingly integral to medical research.

Thank you

As a biostatistician with primary experience in clinical trials, I find the topic of ISCTs both important and challenging. Although I have not worked directly on ISCTs, the manuscript provides insightful examples that illustrate the workflow of ISCTs, highlighting key areas such as modeling and simulation (M&S), verification and validation, and uncertainty quantification. These examples are particularly helpful in understanding the complexities and nuances of ISCT.

However, from a statistical standpoint, my ability to provide in-depth analysis is somewhat limited, as the paper serves primarily as a review and opinion piece rather than a research study with empirical data. It would be beneficial for readers if future work could include more detailed statistical analyses or case studies that demonstrate the practical implementation and challenges of ISCT in medical device development. Such contributions would be invaluable to statisticians and other researchers interested in exploring this emerging field.

Thanks for the comment. We believe that developing statistical methods appropriate for ISCTs (whether novel or adapted from current methods for clinical trials) will be a key task for ISCTs to mature into an established method alongside traditional device evaluation methods. While these biostatistical considerations are outside the scope of the paper, we have added the following paragraph to the discussion section to ensure this important concern is covered and to encourage future research in the area.

- “Clinical trial practice relies on a robust foundation of biostatistics. However, for ISCTs it may be necessary to develop new or adapted statistical methodologies to accurately assess device efficacy and safety. While this paper does not attempt to delve into this complex area, several considerations are noteworthy. These include integrating model uncertainty into the statistical analysis, accommodating the diverse range of possibilities inherent in ISCTs (such as administering both control and therapy for each virtual patient, or simulating multiple interacting therapies in a combinatorial manner), integrating ISCTs with conventional clinical trials (as discussed in [26]), and tailoring protocols specifically for ISCTs, given the distinct factors that constrain them compared to traditional clinical trials. As ISCTs continue to mature, we anticipate that the statistical theory underpinning them will emerge as a burgeoning field ripe for exploration.”

In conclusion, the manuscript successfully highlights the importance and challenges of ISCTs in the current medical landscape. It serves as a foundational piece for further discussion and research in this evolving field, particularly for those of us in the biostatistics community looking to expand our understanding of ISCT methodologies.

<u>Reviewer #3:</u>

Summary:

In this paper, the overall current state of ISCT model approaches and the respective selection/validation/etc. techniques are reviewed. The authors present different levels of validation and credibility assessment (patient, cohort, device, clinician, etc.), and how they are all interconnected. Specific examples of each type of sub-model are and how they can be used for augmenting or replacing clinical trials (in some cases) are provided. Through this review, several important factors are presented for consideration when creating and using an ISCT model, such as errors, variances, and distributions of the cohort(s).

The paper does a good job of presenting existing models and approaches and is successful in explaining the motivation and need for standardized approaches in modeling and simulation. I particularly liked the emphasis on the hierarchical nature of a simulated clinical trial; it was interesting to read about the "building blocks" (in a way) of a virtual cohort.

Thank you

Main points of concern:

My main point of criticism concerns the overall focus of the manuscript and the manuscript’s presentation of the proposed credibility assessment workflow.

By "overall focus" I mean the following comments –

From my perspective, there is an overuse of “refer to this work” or “see [##]” type of sentences and it makes the manuscript seem less cohesive. Many of these instances can be supplemented with the key takeaway(s) from the referenced work, rather than directly referring the reader to other articles.

For instance, in section 2.3.4 (lines 308-309), consider the following statement: “PCLC evaluation provides an example of case C in Figure 1; see e.g., [37], which considers how to generate synthetic

patients from calibrated subject-specific virtual patients." This could be improved by more context regarding [37] and the citation, rather than the "see e.g., [37]" type of redirection. By this, I mean something more like: "PCLC evaluation provides an example of case C in Figure 1; one such example is through {explanation of the cardiovascular responses and methods presented in that conference paper} [37], which considers how to generate synthetic patients from calibrated subject-specific virtual patients."

(My addition/suggestion is within the {} brackets above)

Or, in this statement: "Developing and validating the clinical outcome model may be one of the greatest challenges to demonstrating credibility of an ISCT, due to the difficulty of collecting relevant data; see [12] for a discussion."

It would be nice to provide one or two key takeaways from that discussion in your text, so that the reader can have an idea without having to search for it.

We have gone through the document carefully and addressed this concern. See edits through the document. For example, the ref 37 sentence is now: "For example, [37] defines a method for generating synthetic patients from calibrated subject-specific virtual patients, for a hemorrhage/fluid resuscitation model, that attempts to minimize sampling from non-physiologic parameter space." The ref 12 sentence is now "For example, [12] proposes a method for performing an ISCT in parallel with a real-trial CT, where results from the CT are used to generate the clinical outcome model. The lack of independent data to validate the clinical outcome model under this approach is discussed.". We hope and believe the other citations have been appropriately addressed.

Regarding my comment about the presentation of the proposed example workflow –

I feel as though there isn't enough focus on it, since it is such a shorter section when compared to the rest of the text. I believe that this is one of the core components of this manuscript, but it feels a little lackluster in the main text. While I understand that there is more detail in the supplementary text, I think it would be beneficial to provide more information about its use. If possible, it could be interesting to present a specific example of this workflow being implemented. There are many general examples provided throughout the manuscript (which is a good thing), but the utility of the framework would be more clearly demonstrated if one potential specific implementation is described. For instance, if you presented a start-to-finish application (in an abstract manner) of this workflow regarding the study of a disease or device in a specific population and consider the models that would be needed for that, how they would be validated, etc. in reference to your workflow.

Thank you for this comment. We very much agree - we always believed the workflow was a key novel resource that we are providing in this paper to the community, and it was not ideal that the workflow was relegated to the supplement. This was done to avoid limit the size of the main body text. However, given your comments, and since PLOS Computational Biology does not have a word limit, we have moved the workflow itself from the supplement to Section 4. The revised supplement now just has some comments about how the workflow can be adapted. We believe this modification massively improves the paper by making the workflow prominent and not likely to be missed by readers. In addition, we fully agree that an example of how the workflow would be used would greatly improve the

manuscript, by making the workflow less abstract and more understandable to readers. We have added a high-level hypothetical example, taken from the fields of pacemaker lead durability testing and cardiac mechanics. This is provided as new Section (red text) as bullet points in Section 4. The example is intentionally limited in detail to keep in high-level, but we believe it successfully illuminates the different steps of the workflow and significantly improves the manuscript.

I understand that the execution of the ISCT itself is outside of the paper's scope, but I feel like a little more elaboration in the main text would be useful for contextualization of the workflow.

We had a few sentences about study execution in the discussion but agree it should be discussed early to provide context for the credibility assessment. We now discuss ISCT execution at the end of section 2.2 (which now uses the sentences previously in the discussion) to achieve this.

Figures:

Figure 1 – I think it would be beneficial to also make the border of the "possible components" a dashed line. The light blue is difficult to view at times, so I think that also having a design difference (by changing the border) would be useful in visualizing the flowchart. Additionally, a brief description of the left pane should be included; only the right pane has a description currently.

Done, though we changed the shading of the block rather than making the lines dashed. The figure caption was also edited to include a description of the left-hand side.

Figure 3 – In the caption, there should be a brief description of a), b), and c) and their components, rather than or in addition to "See text discussion for discussion".

Done

Tables:

Table 2 – When providing the examples, I think it would be better to have the example and then the citation. For instance: "MR heating [28, 53], stent structural model [54], heart valve fluid-structure interaction"

Done

Typos and minor comments:

1. Introduction, line 67: The statement "The aims are similar;" seems like it's missing stuff, and the semicolon should be a colon.

Changed to simply "The aims of the present paper are: (i)…"

2. Introduction, lines 70-72: "In this paper our focus is ISCTs for medical devices. However, our conclusions may inform evaluation of ISCTs in other domains, such as ISCTs for pharmaceutical products."

--> I believe that the "." before "However" should be a ";"

Done

3. Introduction, lines 72-76: "This paper complements and builds upon related work such as [10] which provides a theoretical framing for credibility of in silico studies, [11] which discusses limitations of ASME V&V40 2018 for ISCTs, [12] which provides a framework for executing an ISCT using VVUQ concepts, and [13] which considers how to alter ASME V&V40 2018 for ISCTs, including modification of the risk assessment stage which is outside the scope of the present paper."

--> While I understand that this is a list of related work, there are too many "which"s and it becomes difficult to read.

Rephrased to: "This paper complements and builds upon related work, including a theoretical framing for credibility of *in silico* studies [10], a discussion of limitations of ASME V&V40 2018 for ISCTs [11], a framework for executing an ISCT using VVUQ concepts [12], and a proposed alteration to ASME V&V40 2018 for ISCTs, including modification of the risk assessment stage which is outside the scope of the present paper [13]."

4. Introduction, lines 76-78: "The present paper focuses primarily on first-principles models as opposed to data-driven models including machine learning based model (see following section for discussion)."

--> Consider the following rewording: "The present paper focuses primarily on first-principles models as opposed to data-driven models, including those based on machine learning (see following section for discussion)."

Edited to: "The present paper focuses primarily on first-principles models as opposed to data-driven models (including those based on machine learning); see the following section for discussion."

5. Section 2, line 100: there should be a comma between "pathophysiology" and "Those" rather than a period. It sounds disjointed and awkward to read.

Rephrased. New text is: "Requiring a medical intervention in the definition distinguishes ISCTs from simulation studies addressing questions of basic physiology or pathophysiology only, with no medical intervention. Such simulation studies are analogous to observational clinical studies."

6. End of Section 2.2, line 232: no need to redefine "clinical trial (CT)" – similarly, check for other later redefinitions of abbreviations.

Deleted "CT" and checked for any re-definitions and found none (other than in Table 1 which defines acronyms so is a self-contained Table)

7. End of Section 2.3.2, line 278: "in silico" should be italicized

Done

8. Beginning of Section 2.3.6, lines 331-333: The statement "The specific application considered in [26] is cardiac lead fracture, but here we focus on the statistical framework developed in [26] which is the main contribution of that paper." is worded unclearly. It isn't easily apparent that the statistical framework is the main contribution of [26] and it should be reworded.

Done – broken into two sentences. "The specific application considered in [26] is cardiac lead fracture. However, the main contribution of [26] is the statistical framework it provides, and we focus on the framework in this summary."

9. Beginning of Section 2.3.9, line 384: I think the "e.g.," here would read better if it was replaced with "such as" or a similar phrase. Additionally, this whole section is difficult to read because of the many references and should be made easier to follow if possible. The parentheses and such break up the sentences, which makes it so that the reader has to re-read this section multiple times to understand what is being said.

Made the requested edit. We agree the paragraph was difficult to read. We have attempted to make it more readable by introducing bullets and editing the text.

10. Beginning of Section 3.1, lines 407-408: I think that "ISCTs comprise various sub-models" should be "ISCTs are comprised of various sub-models".

Done

11. Next paragraph, lines 416-417: The sentence "In our previous work for patient-specific models [9] we identified unique calculation verification considerations that arise, some of these also apply to ISCTs." is confusing to read and should be reworded.

Re-phrased – split sentence into two. "…we identified unique calculation verification considerations that arise. Some of these considerations also apply to ISCTs."

12. Lines 427-428, when introducing the strategies: I think that "(in no specific order)" might sound better than the existing text "(the following are not ordered by rigor)"

Done

13. Line 439: There should be a semicolon ";" instead of the existing comma "," when introducing the example.

Done

14. Line 471, when describing device model validation: "device/patient model" should be "device-patient model" (which I base off of the later use of "device-patient" in the manuscript)

Done

15. Line 541: "area under receiver operating characteristics (ROC) curve" is later abbreviated as AUC (and ΔAUC), so I would recommend sticking to AUC since ROC is not used again in the text.

Done

16. Section 3.2.3, lines 617-619, when presenting differences in output co-variation: there should be a citation (or citations) for the women having smaller hearts and heart size vs. tissue properties statement.

Done

17. Section 3.3., lines 644-651: I don't think "uncertainties" for each item in this list needs to be repeated because "other sources of uncertainty" was already stated in the text when introducing the list.

Deleted "uncertainties in" at the beginning of each item

18. Discussion, line 729: I think that "device/patient" should be "device-patient"

Done

19. Discussion, line 803: “low-hanging fruit” may be a little too colloquial, I’d suggest an alternative synonym.

Re-phrased the beginning of this sentence. New sentence is “One strategy to achieve these could be ISCTs using patient-specific models that are already approved for clinical use…”
